# Molecular mechanisms underlying the involvement of the sigma-1 receptor in methamphetamine-mediated microglial polarization

**DOI:** 10.1038/s41598-017-11065-8

**Published:** 2017-09-14

**Authors:** Jie Chao, Yuan Zhang, Longfei Du, Rongbin Zhou, Xiaodong Wu, Kai Shen, Honghong Yao

**Affiliations:** 1 Department of Pharmacology, Medical School of Southeast University, Southeast University, Nanjing, China; 2 Department of Physiology, Medical School of Southeast University, Southeast University, Nanjing, China; 3 Institute of Immunology and the CAS Key Laboratory of Innate Immunity and Chronic Disease, School of Life Sciences and Medical Center, University of Science and Technology of China, Hefei, China; 4 Department of Pharmacy, Nantong Tongzhou People’s Hospital, Nantong, China; 5 Institute of Life Sciences, Key Laboratory of Developmental Genes and Human Disease, Southeast University, Nanjing, China

## Abstract

Our previous study demonstrated that the sigma-1 receptor is involved in methamphetamine-induced microglial apoptosis and death; however, whether the sigma-1 receptor is involved in microglial activation as well as the molecular mechanisms underlying this process remains poorly understood. The aim of this study is to demonstrate the involvement of the sigma-1 receptor in methamphetamine-mediated microglial activation. The expression of σ-1R, iNOS, arginase and SOCS was examined by Western blot; activation of cell signaling pathways was detected by Western blot analysis. The role of σ-1R in microglial activation was further validated in C57BL/6 N WT and sigma-1 receptor knockout mice (male, 6–8 weeks) injected intraperitoneally with saline or methamphetamine (30 mg/kg) by Western blot combined with immunostaining specific for Iba-1. Treatment of cells with methamphetamine (150 μM) induced the expression of M1 markers (iNOS) with concomitant decreased the expression of M2 markers (Arginase) via its cognate sigma-1 receptor followed by ROS generation. Sequential activation of the downstream MAPK, Akt and STAT3 pathways resulted in microglial polarization. Blockade of sigma-1 receptor significantly inhibited the generation of ROS and activation of the MAPK and Akt pathways. These findings underscore the critical role of the sigma-1 receptor in methamphetamine-induced microglial activation.

## Introduction

Methamphetamine has a highly addictive effect on the central nervous system (CNS) and is abused throughout the world^1, 2^. Methamphetamine abuse through acute and chronic use is a serious public health problem because of its adverse effects, which include hyperthermia, disruption of the blood-brain barrier, edema and cognitive impairment^3, 4^. Currently, the US Food and Drug Administration (FDA) has yet not approved any pharmacological treatment for psychostimulant dependence, and an effective method of overcoming the negative effects of methamphetamine is urgently needed.

Microglia are resident immune cells and are involved in innate inflammatory responses within the CNS^5^. Microglia are known to be actively involved in various neurological diseases, such as Parkinson’s disease^6^, stroke^7^ and depression^8^. Accumulating evidence suggests that methamphetamine-induced neurotoxicity is associated with microglial activation^9^, and activated microglia are thought to participate in either pro-toxic or protective mechanisms in the brain^10–12^. The classic M1 and alternative M2 phenotypes, which are the two most polarized phenotypes, represent two extremes of a dynamic changing state of microglial activation. M1 and M2 microglia can be distinguished through their expression of a panel of functional and phenotypic markers^13^. ‘M1-like’ activation is characterized by the expression of potent pro-inflammatory mediators such as TNF-α and inducible nitric oxide synthase (iNOS) and is associated with substantial tissue damage. In contrast, ‘M2-like’ activation is associated with the increased secretion of neurotrophic factors and expression of the enzyme arginase 1, which plays a vital role in wound healing^14, 15^. Modulation of microglial phenotype is a well-known and appealing neurotherapeutic strategy, but the molecular mechanisms that drive the methamphetamine-induced switch in microglial phenotype remain poorly understood.

Sigma receptors are classified into two subtypes, sigma-1 and sigma-2 receptors^16^. The sigma-1 receptor, which is a unique ligand-regulated molecular chaperone, is related to many conditions, such as stroke^17^, pain^18^ and HIV infection^19^. The sigma-2 receptor plays a role in pathogenesis by modulating cell proliferation^20^. Earlier studies have also suggested that methamphetamine exhibits significant affinity for the sigma-1 receptor. For example, BD1047, a specific inhibitor of the endoplasmic membrane-bound sigma-1 receptor, reduces neuronal injury in the methamphetamine-exposed hippocampus^21^. A recent study also reported that sigma-1 receptor antagonists attenuate methamphetamine-induced hyperactivity and neurotoxicity^22^. Although the close relationship between the sigma-1 receptor and methamphetamine has been the focus of extensive pharmacological studies, genetic evidence to further elucidate the role and mechanisms of sigma-1 receptor signaling in methamphetamine-mediated microglia activation is still missing.

In the current study, we demonstrate the molecular mechanisms underlying methamphetamine-induced phenotypic changes with a focus on the sigma-1 receptor. This study not only elucidates the cellular signaling mechanisms that underlie methamphetamine-mediated microglial activation but also sheds light on novel therapeutic targets that could be exploited to treat neuroinflammation.

## Materials and Methods

### Reagents

Methamphetamine was purchased from the National Institute for the Control of Pharmaceutical and Biological Products (Beijing, China). The specific MEK1/2 inhibitor U0126, JNK inhibitor SP600125, p38 inhibitor SB203580 and phosphatidylinositol-3 kinase (PI3K) inhibitor LY294002 were purchased from Calbiochem (San Diego, CA). The NADPH inhibitor apocynin was obtained from Sigma-Aldrich (St. Louis, MO, USA), and the STAT3 inhibitor stattic was ordered from Selleck (Houston, TX, US). The concentrations of these inhibitors were based on a concentration-curve study and our previous reports^23^.

### Animals

C57BL/6 N mice (male, 6–8 weeks) were purchased from the Comparative Medicine Centre, Yangzhou University (Yangzhou, China). Sigma-1 receptor knock out (KO) mice were originally obtained from the Laboratory Animal Center of University of Science and Technology of China (Hefei, China) and were backcrossed 10 generations to a C57BL/6 N inbred background. All of the animals were housed under conditions of constant temperature (22 ± 1 °C) and humidity, with a 12 h light (between 8:30 and 20:30)/12 h dark cycle and free access to food and water. After the animals were habituated, the mice were injected i.p. with methamphetamine (30 mg/kg) every 2 h for a total of four injections. Another group of mice received escalating dose methamphetamine. As described in our previous studies^24^, the mice were injected intraperitoneally with incrementally increasing doses on alternating days (i.p, 1.5 mg/kg on days 1–2, once a day; 4.5 mg/kg on days 3–4, once a day; 7.5 mg/kg on days 5–6, once a day; and 10 mg/kg on days 7–8, every 2 h for a total of four times a day). Thirty minutes or twenty-four hours after the last injected, the mice were euthanized and different brain regions were dissected for further analysis of phosphorylation of MAPK/Akt pathways or the expression of iNOS, Arginase and SOCS3, respectively. The control group was injected with saline in the same volume that was used for the methamphetamine treatments. All animal procedures were performed according to the Guidelines of Accommodation and Care for Animals formulated by the Chinese Convention for the Protection of Vertebrate Animals Used for Experimental and Other Scientific Purposes of Southeast University.

### Cell culture

Postnatal (P1 to P3) C57BL/6N mice were purchased from the Comparative Medicine Centre, Yangzhou University (Yangzhou, China). After the membranes and large blood vessels were removed using gauze, dissociated brain tissues were digested with Trypsin-EDTA. The cells were plated in poly-L-lysine-coated cell culture flasks in Dulbecco’s modified Eagle’s medium (DMEM) containing 10% heat-inactivated fetal bovine serum (FBS) and 1% penicillin/streptomycin. The medium was changed for the first time after the cells were allowed to attach for three days. Beginning seven days later, the medium was replaced every 3 days with fresh medium containing 0.25 ng/ml granulocyte/macrophage colony-stimulating factor (GM-CSF) to promote microglia proliferation. The microglia were detached from the flasks by shaking and collected by centrifugation (1500 g × 5 min at 4 °C).

BV-2 immortalized cells were purchased from the China Center for Type Culture collection (Wuhan, China) and were cultured in DMEM supplement with 10% FBS and 1% penicillin/streptomycin and incubated at 37 °C in a humidified atmosphere of 5% CO_2_ and 95% air. The BV-2 cells were used up to passage 20.

### Lentiviral transduction of BV-2 with RFP

BV-2 cells were transduced using LV-RFP lentiviruses (Hanbio Inc., Shanghai, China) as previously described^25, 26^. Briefly, P3–4 BV-2 cells were cultured in 24-well plates at 1 × 10^4^ cells/well in 10% FBS in DMEM for 48 h. The medium was then replaced with 1 ml of fresh medium and 8 µg/ml polybrene. Then, 100 µL of lentivirus solution (10^7^ IU/ml) was added to each well, and the cells were incubated at 37 °C in 5% CO_2_ for 24 h. After the incubation period, the treatment medium was replaced with fresh 10% FBS in DMEM, and the cells were cultured at 37 °C in 5% CO_2_ until they reached >50% confluence. Transduced cells were selected using blasticidin as follows: the medium was replaced with 10 µg/ml puromycin and 10% FBS in DMEM, and the cells were cultured at 37 °C in 5% CO_2_ for 24 h. The cells were then washed twice with fresh 10% FBS in DMEM.

### Reactive oxygen species (ROS) assay

BV-2 cells transduced with RFP-lentivirus were treated with methamphetamine in a time-dependent manner or were pretreated with BD1047 for one h and then treated with methamphetamine. The BV-2 cells were then stained for 30 min with 10 μM carboxy-H2DCFDA (Molecular Probes, Eugene, OR); 1 μM Hoechst 33342 (Molecular Probes, Eugene, OR) was added during the last 5 min of the incubation. After the incubation, the cells were washed with PBS and then immediately visualized using a fluorescence microscope (Zeiss, Göttingen, Germany) or resuspended in PBS containing 20 mM glucose before being analyzed with a Synergy fluorescence plate reader (Bio-Tek Instruments, Winooski, VT). The DCF fluorescence values were divided by the corresponding Hoechst fluorescence values for normalization.

### Western blot

For protein isolation from brain tissue, different brain regions were dissected from mice treated with methamphetamine and homogenized with the lysis buffer according to the manufacture’s instructions. For cell culture, the BV-2 cells were also lysed with the Mammalian Cell Lysis Kit (Sigma-Aldrich, St. Louis, MO, USA). For Nuclear protein extraction, nuclear lysates were isolated using the NE-PER Nuclear and Cytoplasmic Extraction Kit (Pierce, Rockford, IL, USA) according to the manufacture’s instructions. Briefly, treated BV-2 cells were harvested with trypsin-EDTA and centrifuged at 500Xg for 5 min followed by wash with PBS. After centrifuge at 500Xg for 3 min, the cell pellet was resuspended in ice-cold cytoplasmic extraction reagent I with protease inhibitors and incubated on ice for 10 min. Then ice-cold cytoplasmic extraction reagent II was added to the cell suspension and incubated on ice for 1 min with vortex on the highest setting. Once the cytoplasmic portion of the cells was purified, nuclear extraction followed using nuclear extraction reagent containing protease inhibitors.

After protein extraction, the total protein concentration was assessed using the Bradford assay and equal protein samples were loaded on a 12% polyacrylamide gel and transferred to polyvinylidene difluoride membranes, which were then incubated with a blocking buffer containing 5% non-fat dry milk in Tris-buffered saline with Tween-20. The membranes were then incubated with primary antibodies for p-ERK/ERK, p-JNK/JNK, p-p38/p38, p-Akt/Akt, suppressor of cytokine signaling 3 (SOCS3) (Cell Signaling, 1:1000), signal transducer and activator of transcription 3 (STAT3) (Proteintech, 1:1000), iNOS (Abcam, 1:1000) and β-actin (Santa Cruz, 1:500) as previously described^27, 28^.

### Statistical analysis

Statistical analysis was performed using SigmaPlot software (SigmaPlot 11.0, Systat, Inc). The data are expressed as the mean ± SD, and experiments were independently repeated at least three times. The significance of differences between the control samples and those treated with various drugs was tested by one-way ANOVA, with Tukey or Bonferroni correction for multiple comparisons. Values of p < 0.05 were considered significant.

## Results

### Involvement of the sigma-1 receptor in methamphetamine-mediated microglial polarization

**Figure 1:**
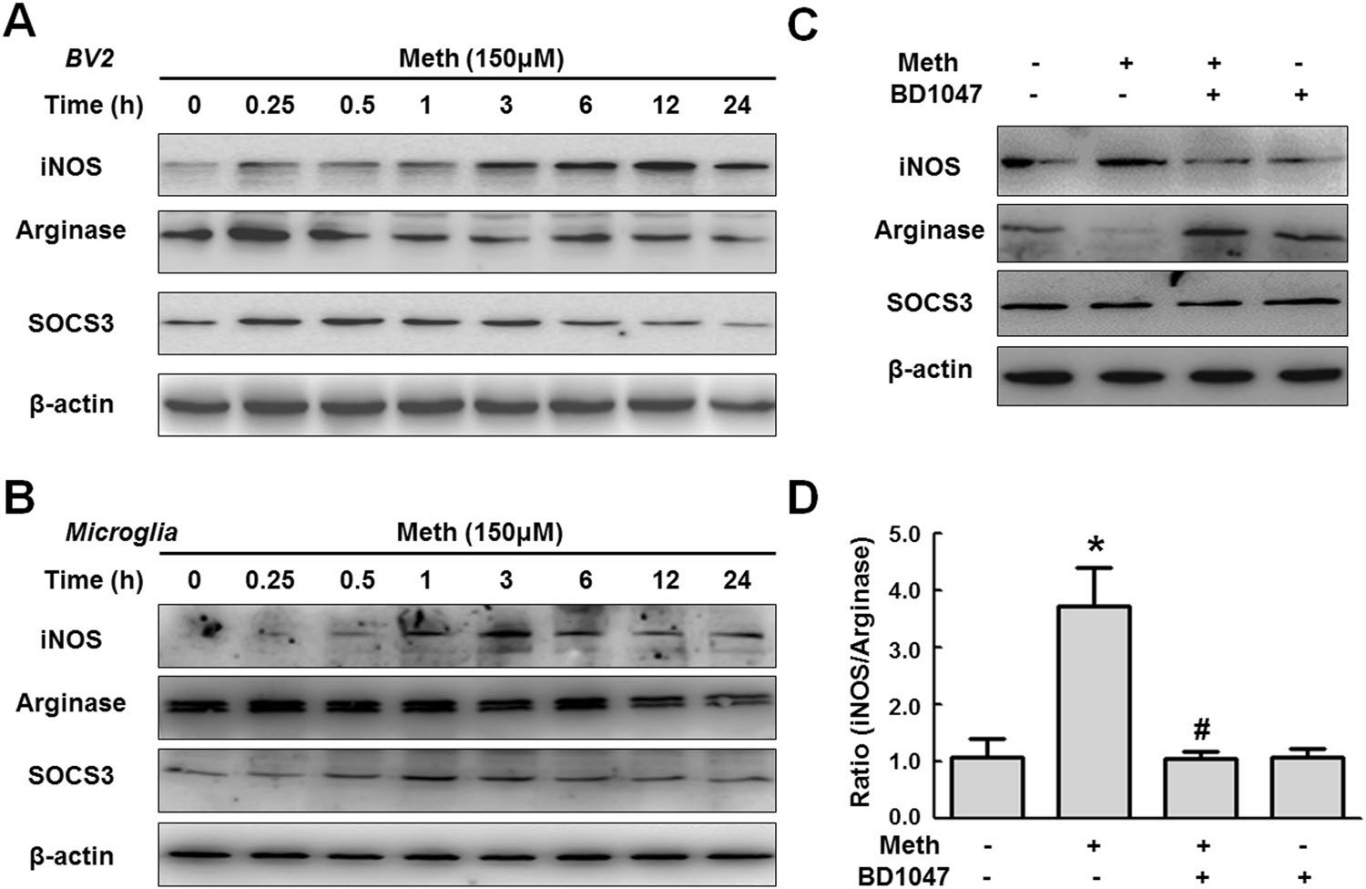
Involvement of the sigma-1 receptor in methamphetamine-mediated microglial polarization. (**A**) Representative western blot showing the effects of methamphetamine (150 μM) on the expression of the M1 polarization markers iNOS, arginase and SOCS3 in BV-2 cells. (**B**) Representative western blot showing the effects of methamphetamine (150 μM) on the expression of the M1 polarization markers iNOS, arginase and SOCS3 in microglia. (**C**) Representative western blot showing the effects of the sigma-1 receptor inhibitor BD1047 on the methamphetamine-induced expression of iNOS, arginase and SOCS3 in BV-2 cells. (**D**) Densitometric analyses for five separate experiments suggest that methamphetamine induced the ratio of M1 marker (iNOS) and M2 marker (arginase), which was attenuated by BD1047 pretreatment. *p < 0.05 vs the control group; ^#^p < 0.05 vs the Meth-treated group using one-way ANOVA. Meth, methamphetamine.

Although recent studies have suggested that BV-2 cells express the sigma-1 receptor, which interacts with methamphetamine at physiologically relevant concentrations^29^, very little is known about the role of the sigma-1 receptor in methamphetamine-mediated microglial polarization. According to our previous study^30^, 150 μM was the optimal dose of methamphetamine to induce glial activation. To assess the time course of methamphetamine’s effects, BV-2 cells were exposed to 150 μM for varying time periods. Treating microglia with methamphetamine increased iNOS expression, however, methamphetamine significantly down-regulated the expression of the anti-inflammatory markers arginase and SOCS3 (Fig. 1A). These findings were also reproduced in primary microglia (Fig. 1B). We next wanted to assess whether the sigma-1 receptor was involved in the methamphetamine-induced phenotypic changes in BV-2 cells. Pretreating BV-2 cells with the sigma-1 receptor antagonist BD1047 for 1 h followed by incubation with methamphetamine for 12 h significantly reduced the methamphetamine-induced increased the ratio of M1 marker (iNOS) and M2 marker (arginase) (Fig. 1C,D). Taken together, these findings clearly demonstrate that the sigma-1 receptor plays a critical role in methamphetamine-induced microglial polarization.

### Methamphetamine induced ROS generation

**Figure 2:**
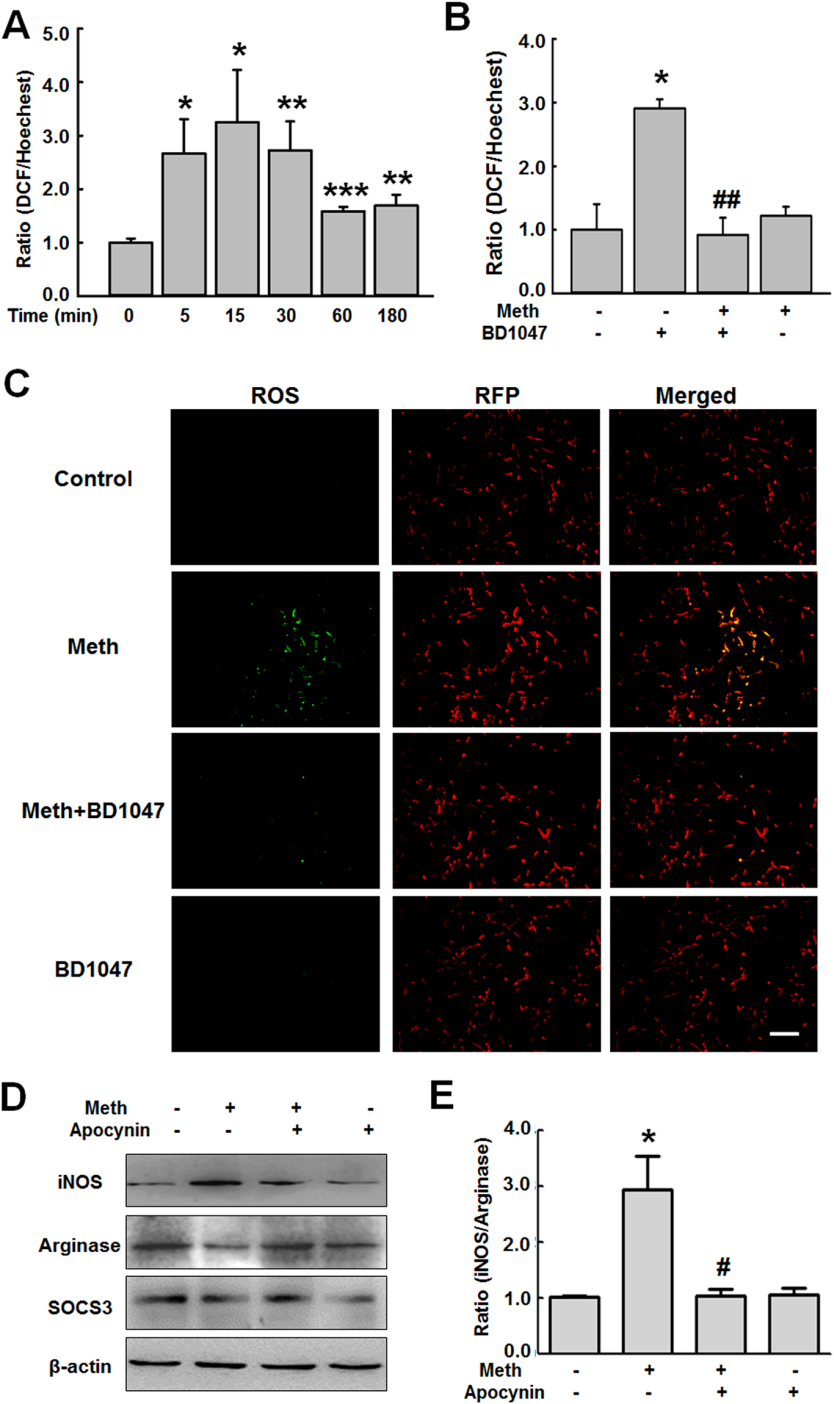
Methamphetamine induced ROS generation. (**A**) Reactive oxygen species (ROS) assay showing that methamphetamine (150 μM) induced ROS generation in a time-dependent manner. *p < 0.05, **p < 0.01, ***p < 0.001 vs the control group using one-way ANOVA. (**B**) ROS assay showing that pretreatment of cells with the sigma-1 receptor inhibitor BD1047 attenuated the methamphetamine-induced ROS generation. *p < 0.05 vs the control group; ^##^p < 0.01 vs the methamphetamine-treated group using one-way ANOVA. (**C**) Representative immunocytochemical images showing that BD1047 attenuated the methamphetamine-induced ROS generation. The ROS level was examined in BV-2 cells transduced with RFP-lentivirus. Green: DCF-DA; Red; RFP-lentivirus. Scale bar: 200 μm. (**D**) Western blot showing that pretreatment with the NADPH inhibitor apocynin attenuated the methamphetamine-induced iNOS, arginase and SOCS3 expression in BV-2 cells. (**E**) Densitometric analyses of five separate experiments suggest that methamphetamine induced the ratio of iNOS and arginase expression, which was attenuated by apocynin pretreatment. *p < 0.05 vs the control group; ^#^p < 0.05 vs the methamphetamine-treated group using one-way ANOVA. Meth, methamphetamine.

While the role of oxidative stress in methamphetamine-induced dopaminergic neurotoxicity is well known^31^, the role of ROS in the mechanisms underlying sigma-1 receptor-mediated modulation of microglial phenotype remains unclear. First, we examined the effect of methamphetamine on the formation of ROS. BV-2 cells exposed to methamphetamine for varying amounts of time exhibited an increase in ROS generation, with a peak at 15 min (Fig. 2A). Since we have determined that ROS also play a crucial role in microglial polarization, we next want to investigate whether there is a link between the sigma-1 receptor and the generation of ROS using a pharmacological approach. Interestingly, pretreating BV-2 cells with BD1047 for 1 h abrogated the methamphetamine-induced ROS formation (Fig. 2B), this was further validated by fluorescence microscope (Fig. 2C). Consistent with the role of the sigma-1 receptor in microglial polarization, in BV-2 cells that were pretreated for 1 h with the NADPH oxidase inhibitor apocynin, differentiation from the anti-inflammatory M2 phenotype to the inflammatory M1 phenotype was also significantly suppressed (Fig. 2D,E).

### Methamphetamine induced activation of the MAPK and PI3K/Akt pathways

**Figure 3:**
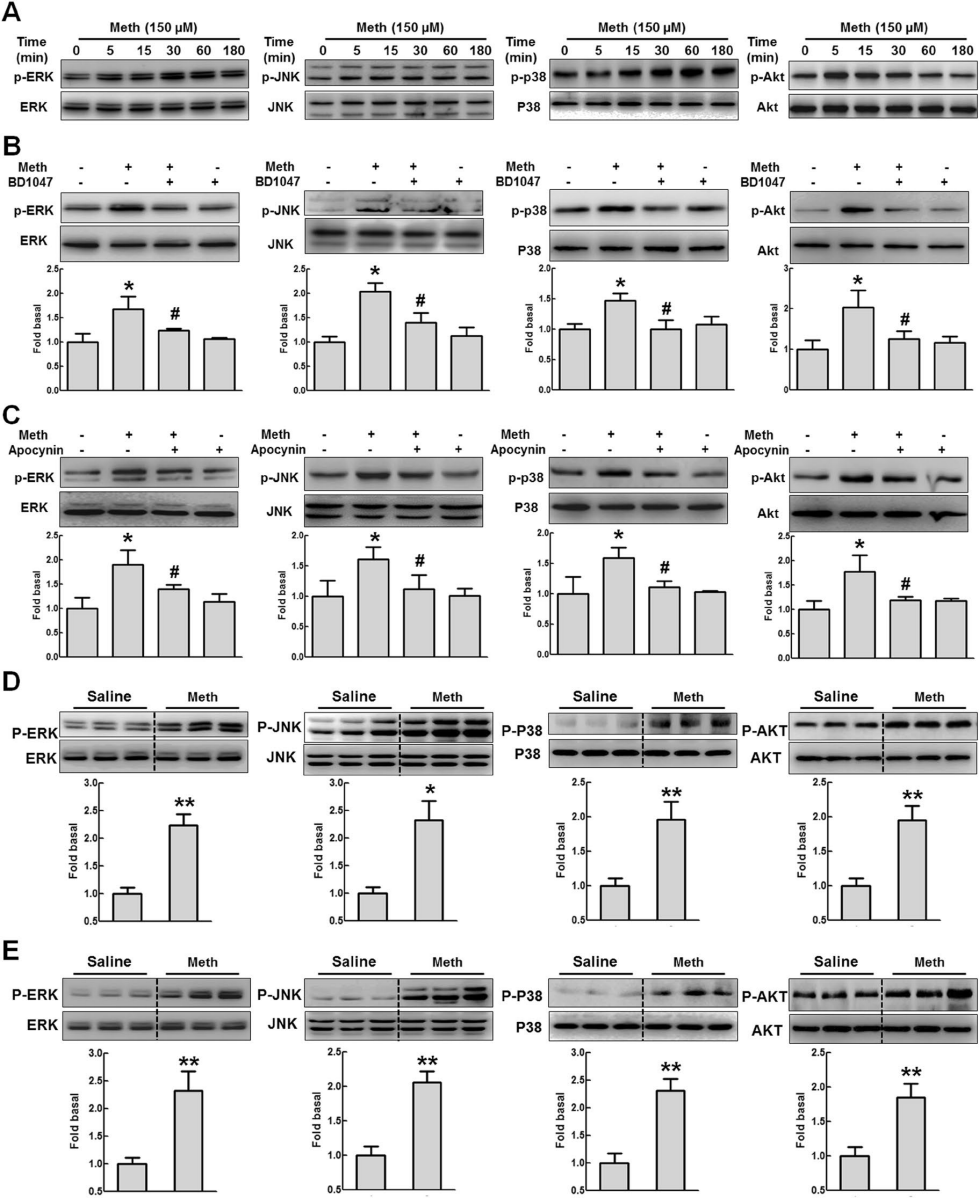
Methamphetamine induced the activation of the MAPK and PI3K/Akt pathways. (**A**) Representative western blot showing the effects of methamphetamine (150 μM) on the phosphorylation of ERK, JNK, p38 and Akt in BV-2 cells. (**B**) Western blot and densitometric analyses showing that pretreatment with the sigma-1 receptor antagonist BD1047 attenuated the methamphetamine-induced phosphorylation of ERK, JNK, p38 and Akt in BV-2 cells. (**C**) Representative western blot and densitometric analyses showing the effects of apocynin on the methamphetamine-induced phosphorylation of ERK, JNK, p38 and Akt in BV-2 cells. *p < 0.05 vs the control group; ^#^p < 0.05 vs the methamphetamine-treated group using one-way ANOVA. (**D**,**E**) Representative western blot and densitometric analyses showed the effects of methamphetamine on the phosphorylation of ERK, JNK, p38 and Akt in the hippocampus. Mice were intraperitoneally injected with methamphetamine (30 mg/kg) every 2 h for a total of four injections (**D**) or with escalating dose methamphetamine (1.5 mg/kg, 4.5 mg/kg, 7.5 mg/kg, and 10 mg/kg) every day for a total of eight days (**E**). Thirty minutes hours after the last injected, the mice were euthanized and the hippocampus was dissected for further analysis of phosphorylation of MAPK/Akt pathways. *p < 0.05, **p < 0.01 vs the control group by Student’s t-test. Meth, methamphetamine.

Several studies have implicated the role of the MAPK and PI3K/Akt pathways in methamphetamine-mediated signaling^32–34^. Treating BV-2 cells with methamphetamine transiently increased the phosphorylation of ERK, JNK, and p38 MAPKs, with a peak at 15 or 60 min (Fig. 3A). Methamphetamine exposure also activated Akt in a time-dependent manner, with maximal activation at 5 min (Fig. 3A). Because sigma-1 receptor-mediated ROS generation and signaling pathway activation are both critical processes that are involved in microglial polarization, we sought to investigate the presence of a link that could tie together the formation of ROS and the signal transduction pathways using a pharmacological approach. BV-2 cells were pretreated with the sigma-1 receptor antagonist-BD1047 or the NADPH oxidase inhibitor-apocynin and then treated with methamphetamine, and activation of the MAPK and PI3K/Akt pathways was assessed. As shown in Fig. 3B,C, pretreatment of cells with BD1047 or apocynin significantly inhibited activation of the MAPK and PI3K/Akt pathways induced by methamphetamine. To better understand the effect of methamphetamine on the activation of the MAPK and PI3K/Akt pathways, mice were intraperitoneally injected with methamphetamine (30 mg/kg) every 2 h for a total of four injections (Fig. 3D) or with escalating dose methamphetamine (1.5 mg/kg, 4.5 mg/kg, 7.5 mg/kg, and 10 mg/kg) every day for a total of eight days (Fig. 3E). Thirty minutes after the last injected, the mice were euthanized and different brain regions were dissected for further analysis of phosphorylation of MAPK/Akt pathways. The administration of methamphetamine significantly increased the phosphorylation of ERK, JNK, p38 and Akt in the hippocampus (Fig. 3D,E). These findings thus demonstrate that sigma-1 receptor-mediated ROS generation occurs upstream of methamphetamine-induced phosphorylation of the MAPK and PI3K/Akt cascades.

### Involvement of the MAPK and PI3K/Akt pathways in methamphetamine-induced translocation of STAT3 into the nucleus

**Figure 4:**
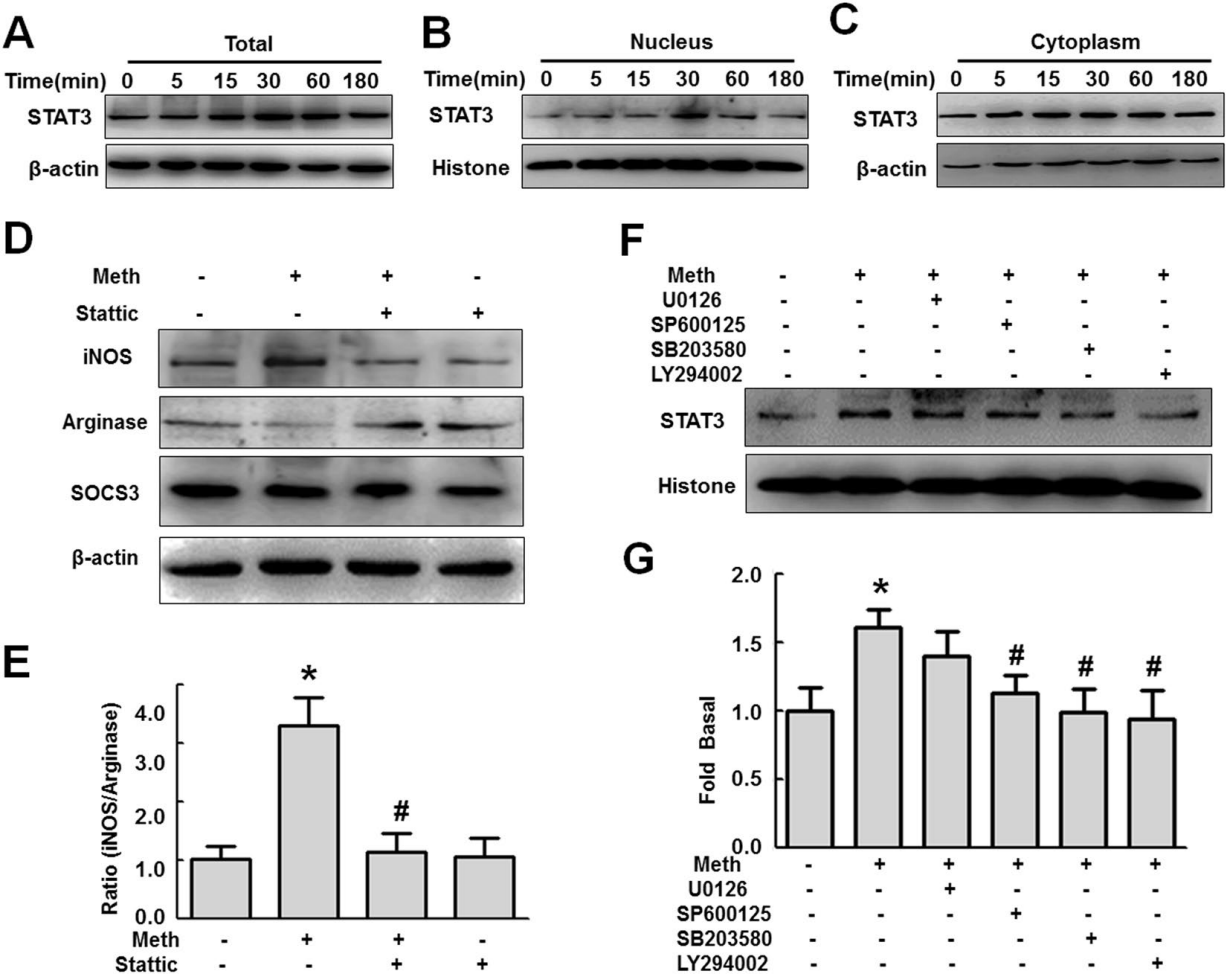
Involvement of the MAPK and PI3K/Akt pathways in the methamphetamine-induced translocation of STAT3 into the nucleus. (**A**–**C**) Representative western blot showing the effects of methamphetamine (150 μM) on the expression of STAT in whole-cell lysates (**A**), nucleus (**B**) and cytoplasm (**C**) of BV-2 cells. (**D**) Representative western blot showing the effects of the STAT inhibitor stattic on the methamphetamine-induced the expression of iNOS, arginase and SOCS3 in BV-2 cells. (**E**) Densitometric analyses of five separate experiments suggest that Meth induced the ratio of iNOS and arginase expression, which was attenuated by stattic pretreatment. (**F**) Representative western blot showing that pretreatment with the ERK inhibitor (U0126, 10 μM), p38 inhibitor (SP600125, 10 μM), JNK inhibitor (SB203580, 10 μM) and Akt inhibitor (LY294002, 10 μM) affected the expression of STAT3 in BV-2 cells. (**G**) Densitometric analyses suggesting that methamphetamine induced STAT3 expression, which was attenuated by pretreatment with SP600125, SB203580 and LY294002 but not U0126. *p < 0.05 vs the control group; ^#^p < 0.05 vs the methamphetamine-treated group using two-way ANOVA. Meth, methamphetamine.

Mounting evidence suggests a link between STAT3 and inflammatory cytokines such as TNF-α and IL-6^35, 36^. Thus, we next sought to examine whether methamphetamine-mediated microglial polarization involved the activation of STAT3. Methamphetamine increased STAT3 expression and concomitantly its translocation into the nucleus, with a maximal response within 30 min (Fig. 4A,B). Also, methamphetamine increased the expression of STAT3 in the cytoplasm (Fig. 4C). Consistent with the results of our studies described above, pretreating BV-2 cells with the STAT3 inhibitor stattic ameliorated the methamphetamine-mediated induction of M1 polarization from M2 phenotype of microglia (Fig. 4D,E). We next wanted to examine the functional role of upstream signaling pathways in the methamphetamine-mediated activation of STAT3. BV-2 cells were pretreated with inhibitors specific for various signaling pathways before they were stimulated with methamphetamine. Intriguingly, the JNK inhibitor SP600125, p38 inhibitor SB203580 and PI3K inhibitor LY294002, but not the MEK1/2 inhibitor U0126, significantly inhibited the methamphetamine-induced translocation of STAT3 (Fig. 4F,G). These findings thus link activation of the MAPK and PI3K/Akt pathways to the downstream translocation of STAT3 as well as to microglial polarization.

### Knockout of the sigma-1 receptor affected methamphetamine-induced microglial activation *in vivo*

**Figure 5:**
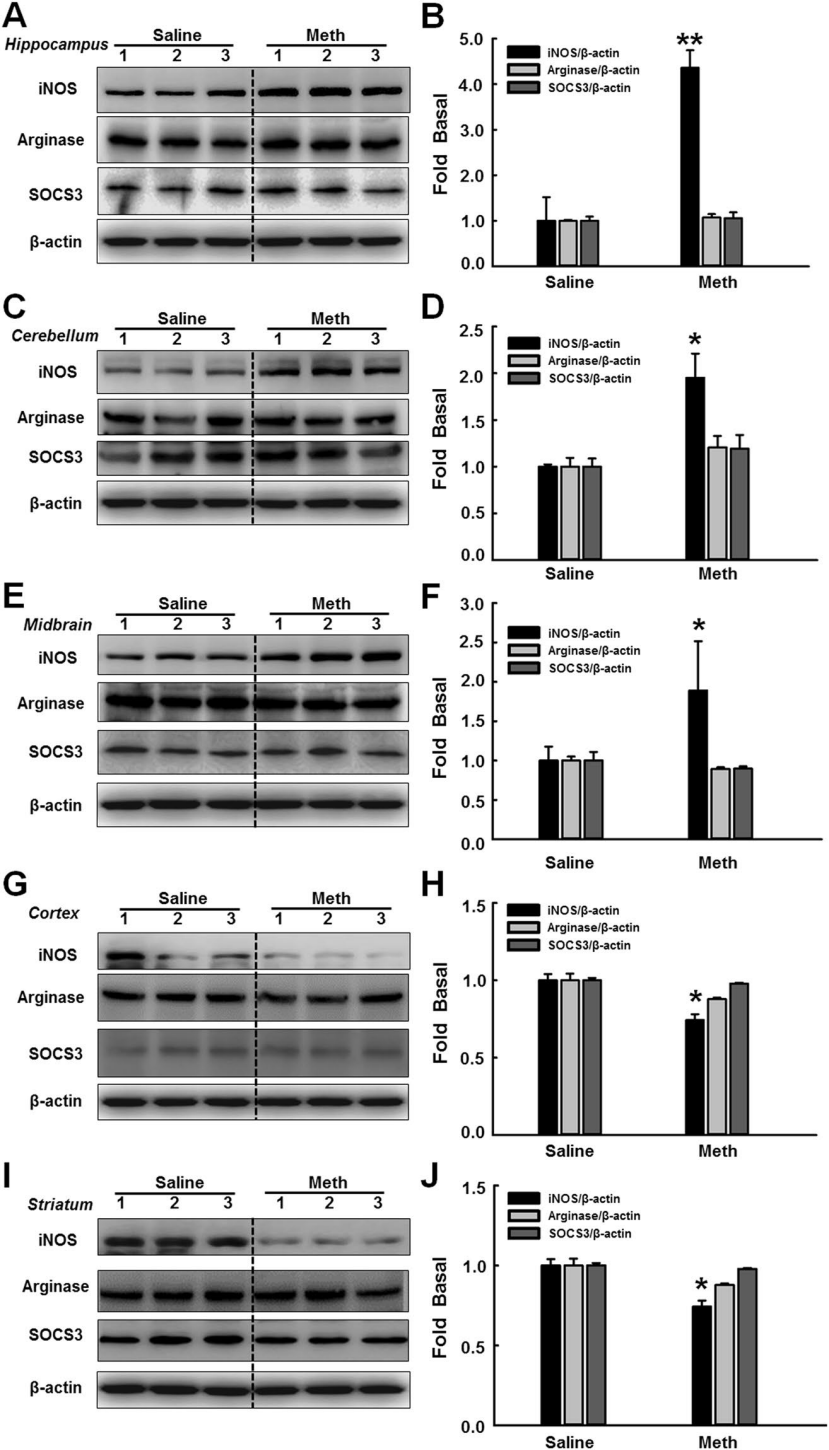
Involvement of the sigma-1 receptor in methamphetamine-induced microglial activation *in vivo*. (**A**–**I**) Representative western blot showing the effects of methamphetamine (30 mg/kg) on the expression of iNOS, arginase and SOCS3 in the hippocampus (**A**), cerebellum (**C**), midbrain (**E**), cortex (**G**) and striatum (**I**). (**B**–**J**) Densitometric analyses suggest that methamphetamine induced iNOS, arginase and SOCS3 expression in the hippocampus (**B**), cerebellum (**D**), midbrain (**F**), cortex (**H**) and striatum (**J**). Mice were intraperitoneally injected with methamphetamine (30 mg/kg) every 2 h for a total of four injections. Twenty-four hours after the last injected, the mice were euthanized and different brain regions were dissected for further analysis of the expression of iNOS, Arginase and SOCS3. N = 5 animals/group. *p < 0.05, **p < 0.01 vs the saline-treated group by Student’s t-test. Meth, methamphetamine.

To better understand the role of the sigma-1 receptor in methamphetamine-induced microglial activation, mice were intraperitoneally injected with methamphetamine (30 mg/kg) every 2 h for a total of four injections. Twenty-four hours after the last injection, the mice were euthanized and different brain regions were dissected for further analysis of the expression of iNOS, Arginase and SOCS3. As an initial screening, we examined the effect of methamphetamine on microglial activation in different brain regions. The administration of methamphetamine significantly increased iNOS expression in the hippocampus, cerebellum, and midbrain (Fig. 5A–C,F–H) and down-regulated iNOS expression in the cortex and striatum (Fig. 5D,E,I,J). The hippocampal findings were consistent with the *in vitro* findings, so this brain region was chosen for all further *in vivo* studies.

**Figure 6:**
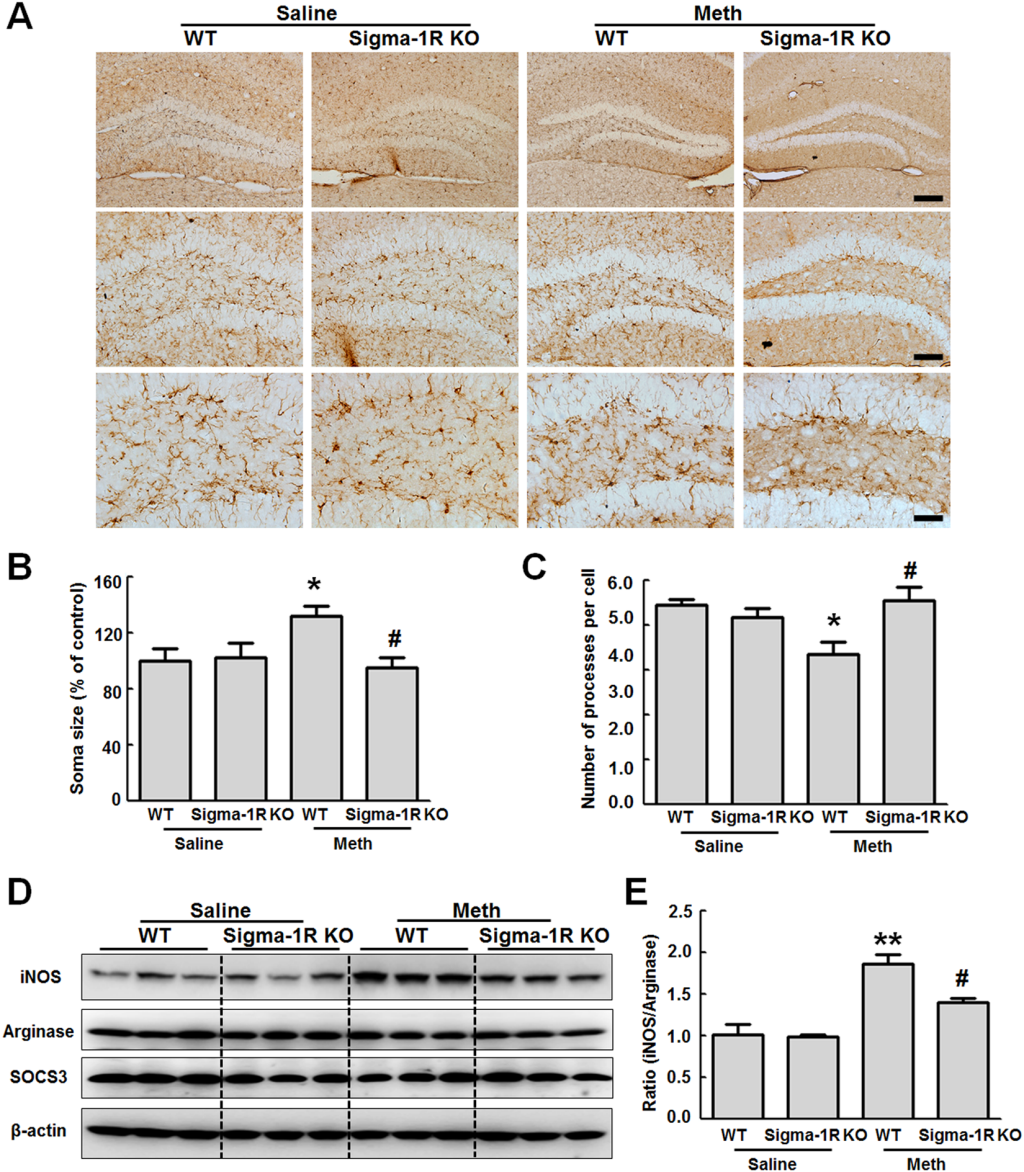
Knockout of the sigma-1 receptor affected methamphetamine-induced microglial activation *in vivo*. (**A**) Methamphetamine induced microglial activation in the hippocampus of WT and sigma-1 receptor KO mice. WT and sigma-1 receptor KO mice were intraperitoneally injected with methamphetamine (30 mg/kg) every 2 h for a total of four injections. Twenty-four hours after the last injected, the mice were euthanized and hippocampal sections were immunostained for Iba-1. Representative image of Iba-1 immunostaining in the hippocampus of mice injected with saline/methamphetamine. Scale bar: 200 μm (upper panel), 100 μm (middle panel), and 50 μm (lower panel). (**B**,**C**) The soma size (**B**) and number of processes per cell (**C**) were quantified in the hippocampus of WT and sigma-1 receptor KO mice. (**D**). Representative western blot showing the effects of methamphetamine (30 mg/kg) on the expression of iNOS, arginase and SOCS3 in WT and sigma-1 receptor KO mice. (**E**) Densitometric analyses suggest that sigma-1 receptor KO mice attenuated methamphetamine-induced increased the ratio of iNOS and arginase expression in WT mice. WT and sigma-1 receptor KO mice were intraperitoneally injected with methamphetamine (30 mg/kg) every 2 h for a total of four injections. Twenty-four hours after the last injected, the mice were euthanized and hippocampal brain region was dissected for further analysis of the expression of iNOS, Arginase and SOCS3. N = 5 animals/group. *p < 0.05, **p < 0.01 vs the saline-treated WT group; ^#^p < 0.05 vs the methamphetamine-treated WT group using one-way ANOVA. Meth, methamphetamine. Sigma-1R: sigma-1 receptor.

In order to examine the role of sigma-1 receptor in microglial activation, microglial activation was examined in methamphetamine-injected sigma-1 receptor KO mice. WT and sigma-1 receptor KO mice were intraperitoneally injected with methamphetamine (30 mg/kg) every 2 h for a total of four injections. Twenty-four hours after the last injected, the mice were euthanized and hippocampal sections were immunostained for Iba-1. As shown in Fig. 6A–C, in the WT group, methamphetamine administration activated microglia in the hippocampus, as demonstrated by the significant increase in microglial soma size and concomitant decrease in cell processes. However, sigma-1 receptor deficiency significantly reduced methamphetamine-induced microglial activation (Fig. 6A–C). In order to further confirm the role of sigma-1 receptor in microglial activation, twenty-four hours after the last injected, the hippocampal brain region was dissected for further analysis of the expression of iNOS, Arginase and SOCS3. As shown in Fig. 6D, methamphetamine also increased iNOS expression in the hippocampus; this increase was significantly ameliorated in sigma-1 receptor KO mice, as quantified in Fig. 6E.

## Discussion

**Figure 7:**
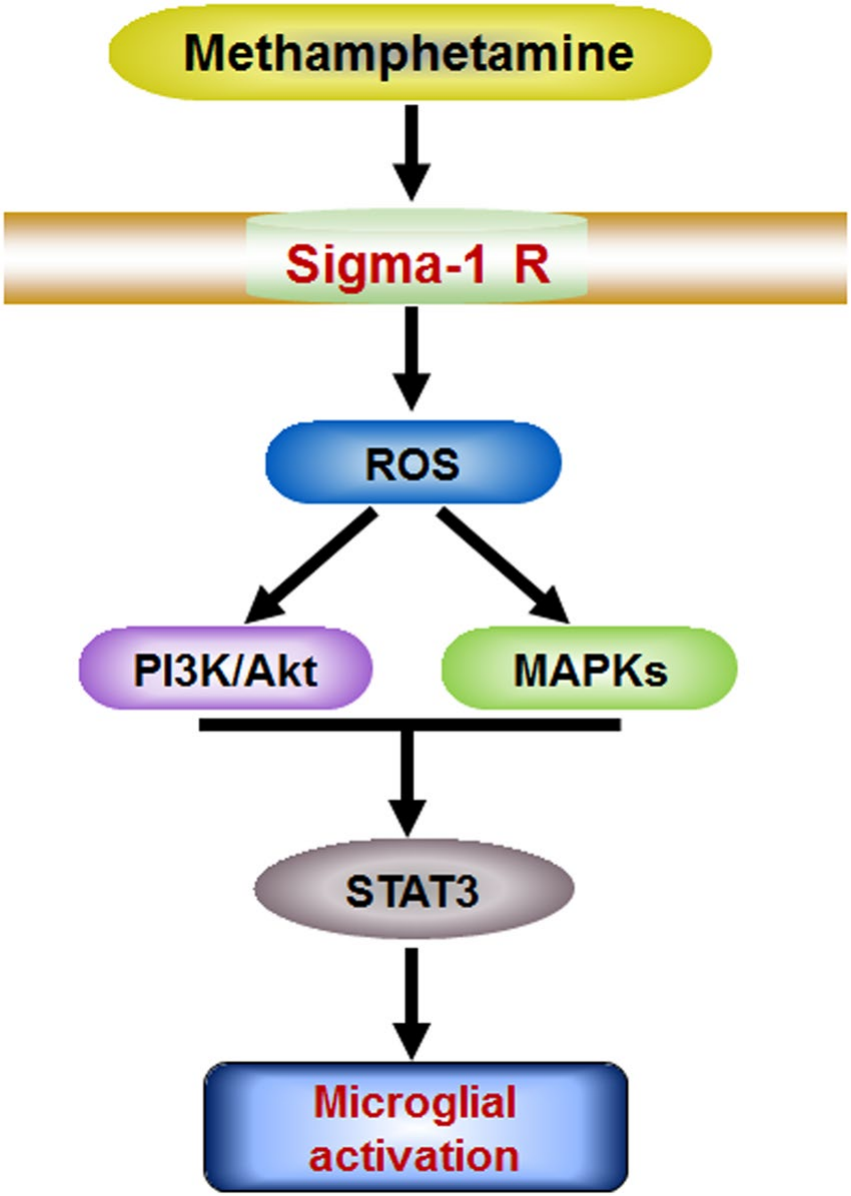
Schematic diagram showing the mechanisms underlying the involvement of the sigma-1 receptor in methamphetamine-induced microglial activation. Meth, methamphetamine. Sigma-1R: sigma-1 receptor.

Although previous studies have demonstrated methamphetamine-induced microglial activation^11, 12, 37^, the molecular and cellular mechanisms involved in this process are not completely understood. Therefore, we demonstrated the vital role of the sigma-1 receptor in methamphetamine-mediated microglial activation *in vitro* and *in vivo*. The findings of the current study also illustrated the detailed mechanisms involved in sigma-1 receptor-mediated microglial activation, which involves ROS generation and the activation of the MAPK and PI3K/Akt pathways. Subsequent translocation of STAT3 then ultimately leads to microglial activation as summarized in Fig. 7.

Sigma receptors, non-opioid receptors, non-phencyclidine receptors, and intracellular receptors modulate multiple signal transduction and neurotransmitter systems. Two subtypes of sigma receptors, sigma-1 and sigma-2, have been identified^38^. The sigma-1 receptor is widely expressed in brain and peripheral tissues and has been localized to the plasma membrane as well as to intracellular structures such as the endoplasmic reticulum^39, 40^. A variety of conditions have been associated with binding affinity for the sigma-1 receptor, including amnesia, schizophrenia^41^, cancer^42^, depression^43^ and addiction^44^. Thus far, the contribution of the sigma-2 receptor remains poorly understood because of the paucity of available experimental tools to study the pathological and physiological processes in which it is involved. Consistent with previous studies^23, 45–47^, inhibiting the sigma-1 receptor with the antagonist BD1047 significantly blocked methamphetamine-induced microglial polarization, implicating the sigma-1 receptor as a promising therapeutic candidate for the neuroinflammatory effects of methamphetamine. In addition, our *in vivo* study, in which we applied a genetic approach using the sigma-1 receptor KO animal model, further confirmed the finding that sigma-1 receptor blockage attenuated methamphetamine-induced microglial activation. However, in contrast to the methamphetamine-mediated microglial polarization we observed in our *in vitro* study, the expression of M2 phenotypic markers was not affected *in vivo*. This difference could be attributed to the fact that only macrophages/microglia were polarized, and this polarization was masked by other cells *in vivo* ^5, 48^. In addition, this difference could also be attributed to differences between the acute and late chronic phase of the methamphetamine model^49^. In this study, we also noticed that there was 50% effect reduced in sigma-1 receptor KO mice, whereas BD1047 abolished the ROS production induced by methamphetamine. This discrepancy maybe due to the fact that: 1) methamphetamine exerted its effect via other targets other than sigma-1 receptor; 2) it is possible for BD1047 to function beyond sigma-1 receptor due to the low specificity as a pharmacological approach.

A growing body of data continues to indicate that ROS play a crucial role in regulating myriad cellular signaling pathways. Moreover, accumulating studies have demonstrated that increased ROS is linked with microglial activation and the secretion of inflammatory factors, such as IL-1β and IL-18^50–52^. One of the mechanisms through which ROS are produced involves a respiratory burst orchestrated by the activation of NADPH oxidase^53^. Consistent with recent findings connecting NADPH oxidase activity with cytokine/chemokine production by microglia^54, 55^, our findings clearly demonstrated that methamphetamine induced NADPH oxidase-generated ROS in a time-dependent manner and that the generated ROS played a critical role in microglial polarization. Interestingly, inhibiting BV-2 cells with BD1047 significantly attenuated the production of ROS induced by methamphetamine. This finding is in agreement with our previous study that showed that the sigma-1 receptor/lipid rafts played a critical role in NADPH-mediated ROS generation in cocaine-exposed microglial cells^23^, suggesting that sigma-1 receptor activation lies upstream of methamphetamine-mediated ROS generation.

We further examined the downstream signaling pathways involved in methamphetamine-induced microglial activation. Our present study revealed the involvement of phosphorylation of ERK, JNK, and the p38 MAPK and PI3K/Akt pathways in microglial activation mediated by methamphetamine. These findings are consistent with the effect of methamphetamine on the activation of the MAPK and PI3K/Akt pathways in human SH-SY5Y cells^33^ and PC12 cells^56^. Mounting evidence suggests that low concentrations of ROS are important for the regulation of pathways such as the TNF-ASK1 pathway^57^. Another key feature of our findings is that the activation of signaling pathways in this process depended on the formation of ROS, as demonstrated by the absence of methamphetamine-induced phosphorylation in the presence of the NADPH oxidase inhibitor apocynin; this result suggests that the activation of signaling pathways occurred downstream of methamphetamine-induced ROS generation.

STATs are involved in a series of biological events, including cell differentiation^58^, angiogenesis^59^, innate immunity^60^ and cell growth regulation^61^. Numerous studies have demonstrated that STAT3 is mainly expressed in glial cells^62, 63^ and is involved in inflammatory reactions^64, 65^. Our current study demonstrated that methamphetamine exposure induced time-dependent up-regulation of STAT3 protein and translocation of STAT3 into the nucleus. Similar to the results of our previous study described above, STAT3 blockage significantly ameliorated methamphetamine-mediated microglial activation. Further dissection of the signaling pathways involved in the methamphetamine-mediated translocation of STAT3 using a pharmacological approach revealed the involvement of the JNK, p38 and PI3K/Akt pathways but not of ERK. This finding is consistent with a previous study that showed that methamphetamine induced ERK phosphorylation; unfortunately, however, specific blockade of the ERK pathway failed to rescue the methamphetamine-induced death of SH-SY5Y cells^33^. Collectively, these results demonstrate that the MAPK and PI3K/Akt pathways lie upstream of STAT3.

Microglial activation is known to be a key feature of neuroinflammatory processes^66, 67^. Activated microglia are characterized by a dynamic changing state, namely the classic M1 and alternative M2 activation. Our present study demonstrated that M1-M2 microglial polarization is a tightly controlled process that involves a set of signaling pathways and transcriptional regulatory networks. Our data provide the first evidence that the sigma-1 receptor plays a key role in methamphetamine-mediated microglial activation through the use of a genetic approach.

In summary, we detailed the molecular mechanisms associated with the dynamic changes involved in microglial polarization and provided evidence of their interactions; understanding of these interactions is crucial to elucidate the molecular basis of methamphetamine-induced disease progression and to design novel microglia-mediated therapeutic strategies.

### Declaration:

 The use of cell and animal was performed in accordance with the approved guidelines of the Research and Development Committee of Southeast University.
