# Author Correction: Molecular mechanisms underlying the involvement of the sigma-1 receptor in methamphetamine-mediated microglial polarization

**DOI:** 10.1038/s41598-021-82645-y

**Published:** 2021-03-11

**Authors:** Jie Chao, Yuan Zhang, Longfei Du, Rongbin Zhou, Xiaodong Wu, Kai Shen, Honghong Yao

**Affiliations:** 1 Department of Pharmacology, Medical School of Southeast University, Southeast University, Nanjing, China; 2 Department of Physiology, Medical School of Southeast University, Southeast University, Nanjing, China; 3 Institute of Immunology and the CAS Key Laboratory of Innate Immunity and Chronic Disease, School of Life Sciences and Medical Center, University of Science and Technology of China, Hefei, China; 4 Department of Pharmacy, Nantong Tongzhou People’s Hospital, Nantong, China; 5 Institute of Life Sciences, Key Laboratory of Developmental Genes and Human Disease, Southeast University, Nanjing, China

Correction to: *Scientific Reports* 10.1038/s41598-017-11065-8, published online 14 September 2017

This Article contains errors in Figure 1 and Figure 5.

In Figure 1, the western blot images of iNOS in Figure 1A and β-actin in Figure 1B are incorrect. The correct Figure 1 appears below as Fig. 1.

**Figure 1:**
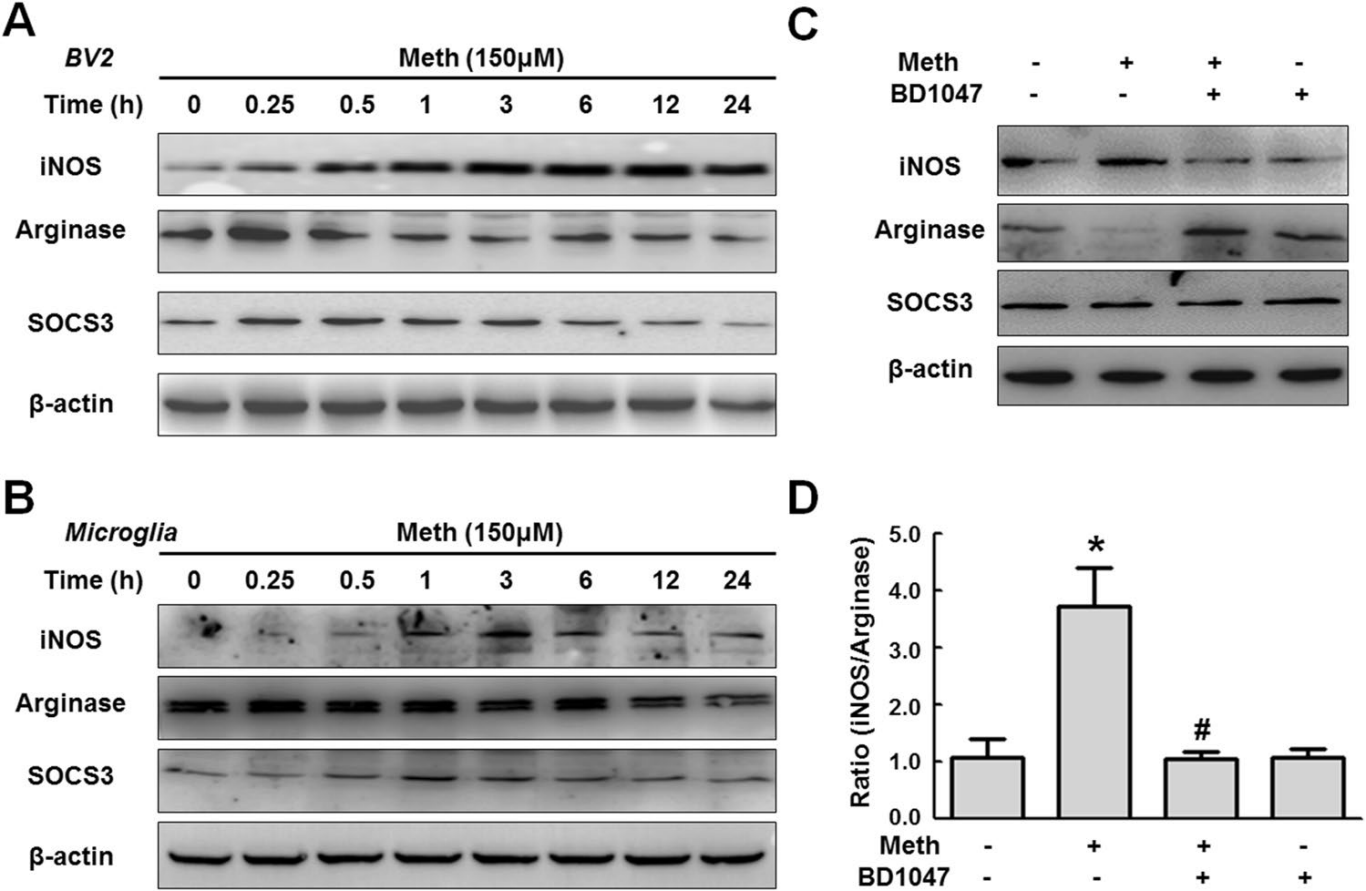
A correct version of the original Figure 1.

In Figure 5, the bar graph in Figure 5H is presented repeatedly by Figure 5J inattentively. The correct Figure 5 appears below as Fig. 2.

**Figure 2:**
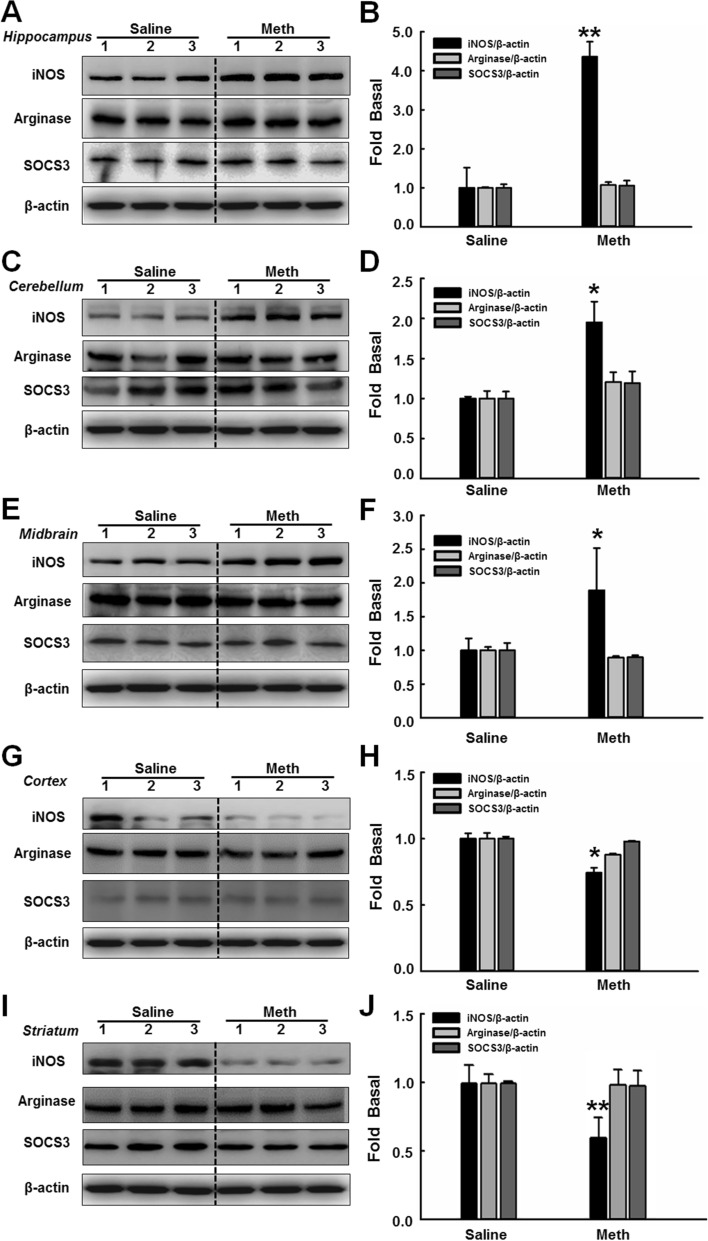
A correct version of the original Figure 5.

Consequently, in the Results section, under ‘Knockout of the sigma-1 receptor affected methamphetamine-induced microglial activation *in vivo*’,

“The administration of methamphetamine significantly increased iNOS expression in the hippocampus, cerebellum, and midbrain (Figure 5A–C,F–H) and down-regulated iNOS expression in the cortex and striatum (Figure 5D,E,I,J).”

should read:

“The administration of methamphetamine significantly increased iNOS expression in the hippocampus, cerebellum, and midbrain (Figure 5A–F) and down-regulated iNOS expression in the cortex and striatum (Figure 5G–J).”

